# Nematicide influence on cotton yield and plant-parasitic nematodes in conventional and sod-based crop rotation

**DOI:** 10.21307/jofnem-2020-034

**Published:** 2020-04-16

**Authors:** Lesley A. Schumacher, Zane J. Grabau, David L. Wright, Ian M. Small, Hui-Ling Liao

**Affiliations:** 1 Entomology and Nematology Department, University of Florida, Gainesville, FL, 32611; 2 North Florida Research and Education Center, University of Florida, Quincy, FL, 32351

**Keywords:** Cotton, Crop rotation, Fluopyram, *Helicotylenchus*, Management, *Mesocriconema*, Nematicide, Plant-parasitic nematodes, Reniform nematode, Ring, *Rotylenchulus reniformis*, Spiral

## Abstract

Plant-parasitic nematodes (*Rotylenchulus reniformis* (reniform, RN), *Helicotylenchus dihystera* (spiral), and *Mesocriconema ornatum* (ring)) and yield were investigated in cotton phases of conventional (peanut–cotton–cotton) and sod-based (bahiagrass–bahiagrass–peanut–cotton) rotations with or without irrigation and fluopyram nematicide at a long-term research site, established in 2000, in Quincy, Florida, USA. Objectives were to determine impacts of nematicide application on cotton yield and evaluate effects of nematicide on plant-parasitic nematodes in these rotations in 2017 and 2018. Reniform nematode population densities were greater in conventional cotton than sod-based cotton. Ring and spiral nematode population densities were greater in sod-based cotton than conventional cotton. Plots receiving nematicide had increased RN population densities in preplant 2018 soil samples and spiral nematode population densities in preplant 2017, harvest 2017, preplant 2018, and harvest 2018 soil samples compared to untreated plots. Cotton seed yield in conventional rotation was increased by 18% following nematicide application in 2017 but decreased by 10% in sod-based rotation in 2018, relative to the untreated control. Sod-based rotation had greater cotton yield than conventional rotation in 2017 and 2018. Nematicide application did not improve cotton yield in sod-based rotation and was inconsistent in conventional rotation.

Cotton (*Gossypium hirsutum*) is an important agronomic crop in the Southeastern United States. In 2018 cotton seed production in the United States was estimated at 5.11 million metric tons with an average yield of 1.24 metric tons per hectare (NASS-USDA, 2019). Reniform nematode (*Rotylenchulus reniformis* Linford and Oliveira, RN) is a significant pathogen of cotton that is typically managed by crop rotation or nematicide application (Linford and Oliveira, 1940; Moore and Lawrence, 2012). Reniform nematode is a sedentary endoparasite that reduces yield, boll size, and delays maturity (Robinson et al., 1997; Khanal et al., 2018). Cotton is considered one of the most severely affected hosts of RN, with possible yield losses of 60% and fall population densities of 1,000 RN/100 cm^3^ soil estimated as a damage threshold (Blasingame et al., 2002; Doshi et al., 2010; Moore and Lawrence, 2012). Aboveground damage is visible as waves of uneven cotton plant growth that may include wilting, stunting, and chlorosis (Lawrence and McLean, 2001; Blasingame et al., 2002; Robinson, 2007).

Rotating to a non-host crop may provide short-term suppression of plant-parasitic nematodes in cotton production (Starr et al., 2002). For instance, crop rotation to a non-host like peanut (*Arachis hypogaea*) or corn (*Zea mays*) is an effective means to manage RN (Moore and Lawrence, 2012). Rotation to a non-host for one or more years can reduce RN populations below economic thresholds into the subsequent cotton crop (Stetina et al., 2007; Moore and Lawrence, 2012). One conventional crop rotation in the Southeastern United States consists of peanut followed by two years of cotton. An alternative, sod-based rotation (SBR) uses at least two years of perennial bahiagrass (*Paspalum notatum*) followed by subsequent peanut and cotton. Including bahiagrass into the rotation reduces pathogen pressure and builds organic matter, which aids in soil water holding capacity as well as improving other soil properties (Katsvairo et al., 2006, 2007). SBR has been shown to produce high yields with reduced amounts of fertilizer, pesticide, and irrigation (Norden et al., 1977; Wright et al., 2010; Dourte et al., 2015). Prior research suggests SBR may be beneficial for managing RN during peanut phases, but SBR impacts on RN during cotton phases have not been examined (Tsigbey et al., 2009).

Nematicide application is another primary method to manage plant-parasitic nematodes in cotton (Moore and Lawrence, 2012; Khanal et al., 2018). Several studies have shown that plant-parasitic nematode populations decrease after nematicide treatments in horticultural crop production (Carrascosa et al., 2014; Coleman and Wall, 2015). However, there is a desire to minimize nematicide application because of cost and potential negative environmental impacts associated with its use (Khanal et al., 2018). Fluopyram is the active ingredient in a nematicide recently made available in cotton production. Most nematicides work by disrupting chemoreception and nervous system function in nematodes (Haydock et al., 2006). Fluopyram selectively inhibits Complex II of the mitochondrial respiratory chain in the mitochondria of nematodes, which results in immobility and death (Heiken, 2017). In the field, *Meloidogyne incognita* populations were reduced and tomato root growth enhanced after fluopyram application (Ji et al., 2019). Additionally, fluopyram increased lint yields in fields with high *Meloidogyne incognita* pressure (Roper, 2017). In the laboratory, RN infectivity in tomato roots was successfully reduced after fluopyram application (Faske and Hurd, 2015). However, this product is nematistatic because its effects can be reversed after a certain period of exposure (Faske and Hurd, 2015). While effective in managing *Meloidogyne* spp. on vegetables in greenhouse and microplot studies (Hajihassani et al., 2019; Silva et al., 2019), evaluation of fluopyram effects on other plant-parasitic nematodes is needed in various rotation systems.

Little published research exists on fluopyram effects on RN in cotton in the field since it is a relatively new nematicide. To address this paucity of information, there is a need to investigate fluopyram performance in conjunction with crop rotation in cotton because nematicide protection from yield loss may vary based on the population densities of the different plant-parasitic nematode genera present. If SBR sufficiently manages RN, applying nematicide may result in minimal yield impact, which may allow growers to eliminate nematicide application when using SBR. We hypothesize that fluopyram reduces populations of all plant-parasitic nematodes in both conventional cotton and SBR schemes. Previous studies showed that RN pressure was greater in conventional cotton than SBR at the site (Tsigbey et al., 2009). Therefore, we further hypothesize that yield impact of nematicide application will be greater in conventional rotation than SBR due to greater nematode pressure in conventional rotation than SBR.

Water management, such as irrigation, is used extensively in agriculture and can also influence nematodes. Water used in agricultural systems is responsible for 70% of freshwater use globally (FAO, 2007). Moore et al. (2010) showed that irrigation had no effect on the migration of RN females and juveniles, but males migrated faster in the presence of irrigation. Furthermore, RN can survive for long periods in an anhydrobiotic state (Birchfield and Martin, 1967). However, RN is not as well adapted to anhydrobiosis as organisms found in more stressed (i.e. drier) environments (Womersley and Ching, 1989). There is a need for more information on the impact of irrigation on plant-parasitic nematode populations in various agroecosystems and how this relates to other cultural methods like crop rotation. SBR reduces the need for irrigation in the subsequent crop due to larger roots of following crops, so comparing irrigation and non-irrigation in both conventional and SBR is of interest from an agronomic perspective as well (Katsvairo et al., 2006).

Main objectives for this research were to: (i) assess if SBR helps manage plant-parasitic nematodes by reducing population densities and increasing crop yields; (ii) determine if the addition of the fluopyram nematicide further reduces plant-parasitic nematodes in conventional and SBR; and (iii) determine if yield impacts of nematicide application vary by crop rotation system. Further objectives evaluated the effect of irrigation on plant-parasitic nematode population densities.

## Material and methods

### Study site

Studies were conducted at the University of Florida’s North Florida Research and Education Center (NFREC) in Quincy, FL (30°32.79’N, 84°35.50’W). A sod-based rotation study has been in place at this long-term agricultural research site since 2000 (Zhao et al., 2010). The soil was a Dothan sandy loam (fine-loamy, kaolinitic, thermic Plinthic Kandiudult) with 85% sand, 5% silt, and 10% clay (Zhao et al., 2010). The site was naturally infested with RN, spiral (*Helicotylenchus dihystera*), and ring (*Mesocriconema ornatum*) nematodes (Tsigbey et al., 2009).

### Experimental design

The study used a complete block design with a modified split-split plot arrangement (irrigation by crop rotation phase by nematicide) with three replicates. The research site includes four-year bahiagrass–bahiagrass–peanut–cotton (sod-based rotation, SBR) rotation and a three-year peanut–cotton–cotton (conventional) rotation where each crop phase of each rotation is present each year were compared based on previous research (Katsvairo et al., 2007). Of these, the three cotton crop phases were sampled for this study in 2017 and 2018, two from the conventional rotation (first and second-year cotton) and one from SBR (Table 1). Therefore, subplot treatments were first-year conventional cotton (C1), second-year conventional cotton (C2), and sod-based cotton (CS). All rotation phases were represented each year, with and without irrigation. Irrigation (main plot treatment) was supplied via a lateral line overhead system and applied to half of each replicate as necessary while the other half was rainfed only. Velum^®^ Total nematicide (fluopyram and imidacloprid, Bayer Crop Science, Research Triangle Park, NC) was the sub-subplot treatment and all three cotton phases received this treatment in 2017 and 2018. The first half of the sub-sub plots received nematicide in-furrow at planting via a two-row tractor-driven Monosem planter (Monosem Co., Edwardsville, KS) using 8002 flat fan nozzles placed perpendicularly to the row spraying just before the seed was dropped into the furrow at a rate of 1.3 l/ha (0.24 kg a.i./ha) while the second half of the sub-subplots received no nematicide. The in-furrow spray was stopped before continuing to sub-subplots receiving no nematicide. Sub-subplots were 1.8 m by 9.1 m (10 rows of cotton). Sub-subplots planted to cotton (*n* = 36) in 2017 and 2018 were assessed.

**Table 1.** Crop phase for bahiagrass, peanut, and cotton during 2016 to 2019^a^.

Phase number	Rotation	2016	2017	2018	2019
1	Conventional	P	*C1*	*C2*	P
2	Conventional	C1	*C2*	P	C1
3	Conventional	C2	P	*C1*	C2
4	Bahiagrass	CS	B1	B2	PS
5	Bahiagrass	B1	B2	PS	CS
6	Bahiagrass	B2	PS	*CS*	B1
7	Bahiagrass	PS	*CS*	B1	B2

**Notes:** aC1 and C2 are first and second-year conventional cotton, respectively. CS is sod-based cotton. P and PS are conventional and sod-based peanut, respectively. B1 and B2 are first and second-year bahiagrass, respectively. Phases in italic were included in the study in 2017 and 2018.

### Trial maintenance

Detailed site maintenance information is provided in Table 2. Aside from crop rotation, irrigation, and nematicide treatments described above, planting and harvest were uniform across the site. The site used strip tillage and a winter cover crop of oats planted in December of each year (terminated in March) in the peanut and cotton rotations. A preplant 5-15-30 (N-P-K) fertilizer was applied at 280.2 kg/ha based on previous site recommendations. Additionally, all subplots received 27.2 kg N. Deltapine^®^ 1646B2XF cotton was planted using a two-row Monosem planter at the rate of 13 seeds/m of row. The standard insecticide, herbicide, and growth regulators were applied uniformly as needed. Plots received irrigation at 1.5 cm per irrigation event. Cotton was harvested using a two-row Case IH (CNH Industrial America, LLC, Racine, WI) cotton picker from the third, fourth, seventh, and eighth rows of each plot and weighed (two weights per plot). Lint and seed yield were then calculated by ginning a 0.9 kg subsample from each plot.

**Table 2.** Cotton planting, nematode sampling, cotton harvest, and irrigation dates in 2017 and 2018.

	2017	2018
Cotton planting	April 28	April 27
Soil sampling^a^		
Preplant (Pi)	April 13	April 19
Midseason (Pm)	June 19	June 22
Harvest (Pf)	September 25	September 25
Root sampling	June 19	June 22
Cotton harvest	October 17	October 8
Irrigation events^b^	May 10	May 12
	July 5	June 26
	August 2	July 12
	August 25	July 18

**Notes:** ^a^Pi, Pm, and Pf are nematode sampling events before planting, at midseason (52 and 56 days after planting in 2017 and 2018), and at harvest (150 and 151 days after planting in 2017 and 2018), respectively. ^b^Amount of water per irrigation event was 1.5 cm.

### Soil sampling and nematode quantification

Soil samples from the two center rows of each plot (8 cm or less away from plants) were collected to a 30 cm depth using an Oakfield tube before planting, at midseason (52 and 56 days after planting in 2017 and 2018, respectively) and at harvest (150 and 151 days after planting in 2017 and 2018, respectively). In total, 12 cores were taken per plot and mixed to achieve a composite soil sample. Samples were stored at 4°C for less than three days before processing. Prior to extraction, soil samples were sieved through a screen with 0.64 cm apertures to achieve a more uniform soil particle size. Nematodes were extracted from 100 cm^3^ soil using a modified sucrose-centrifugation method (Jenkins, 1964).

Nematode samples were fixed in 2% formalin prior to identification. Nematodes were counted from soil samples using a 400× inverted microscope (Carl Zeiss Inc., Thornwood, NY) and identified morphologically. Total nematode population density was recorded, the first 200 nematodes encountered identified to genus based on a key by Mai and Mullin (1996), and then adjusted to the absolute abundance per 100 cm^3^ by adding up totals from each nematode genus present. Of the plant-parasitic nematode genera encountered, only RN, spiral, and ring nematodes were statistically analyzed. Furthermore, RN males were enumerated separately from immature females and juveniles. The ratio of RN immature females and juveniles to males was statistically analyzed. Reproduction factor (Pf/Pi) was also calculated for each plant-parasitic nematode in 2017 and 2018.

### Statistical analysis

Nematode data were analyzed separately for each sampling date. Each variable within each sampling date of 2017 and 2018 (preplant, midseason, and harvest) was analyzed using three-way, split-split plot ANOVA in R version 3.3.1 (The R Foundation for Statistical Computing, Vienna, Austria). ANOVA models were checked for homogeneity of variances using Levene’s test and normality of residuals checked graphically (Levene, 1960; Cook and Weisburg, 1999). If there were significant (*p* ≤ 0.05) interactions (irrigation by crop or crop by nematicide) in the full ANOVA, these interactions were analyzed rather than main effects of crop, nematicide, or irrigation. Nematicide effects for each crop phase (C1, C2, and CS) were analyzed separately if crop by nematicide interaction was significant (*p* ≤ 0.05). Crop effects for each irrigation treatment were analyzed separately if irrigation by crop interaction was significant (*p* ≤ 0.05). For variables with significant (*p* ≤ 0.05) crop or nematicide effects, treatment means were separated using Fisher’s least significant difference (LSD) test (*p* ≤ 0.05).

## Results

### RN populations

**Figure 1:**
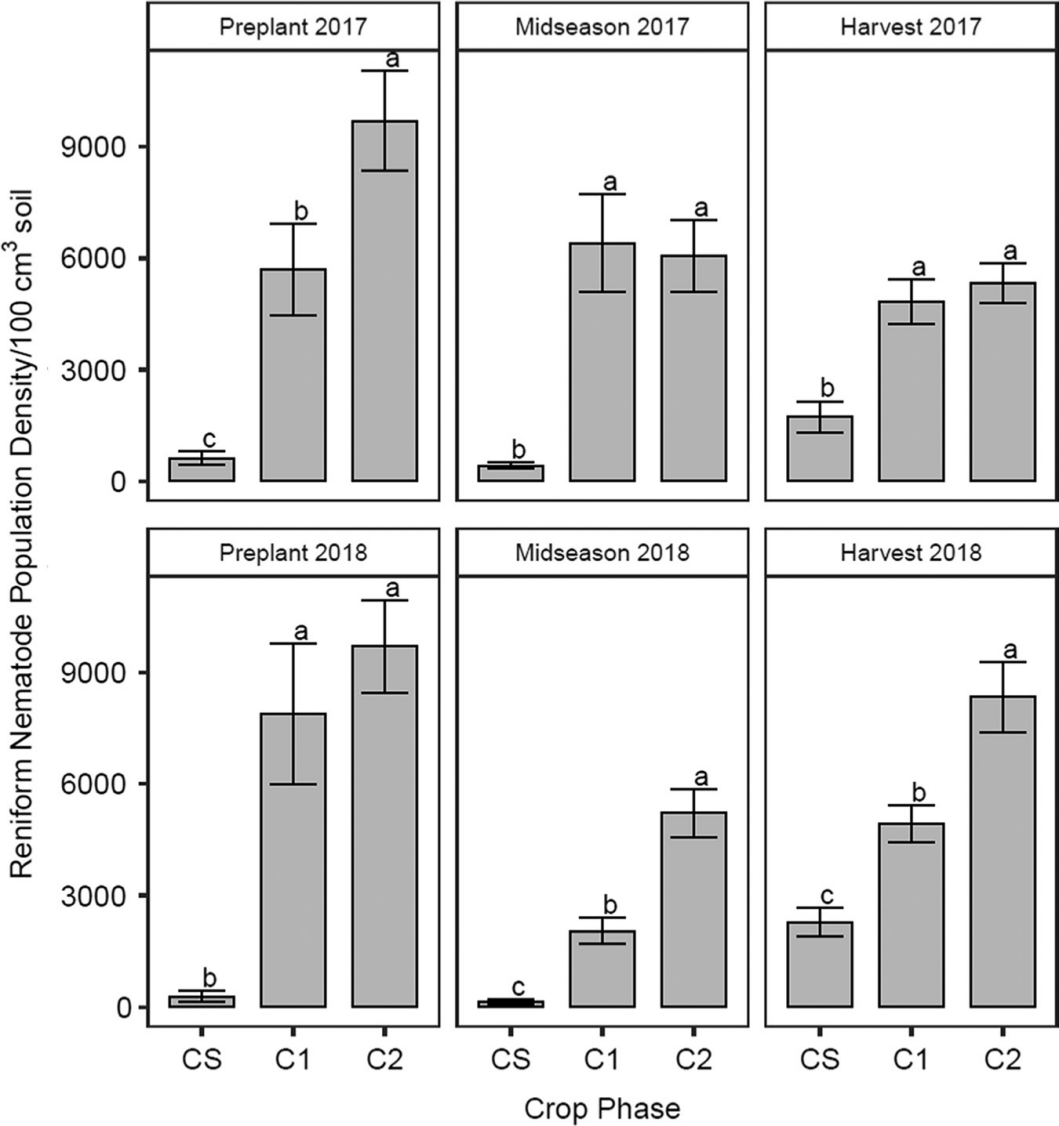
Reniform nematode (*Rotylenchulus reniformis*) population density (nematodes/100 cm^3^ soil) as influenced by crop phase in 2017 and 2018 soil samples. CS is the cotton phase of the sod-based rotation, C1 is first-year conventional cotton, and C2 is second-year conventional cotton. Different letters denote significant differences between crop phases (Fisher’s protected LSD, *p* ≤ 0.05).

Crop rotation significantly affected total RN population density in all seasons (Fig. 1, Table 3). RN populations were greatest in C2 and least in CS in preplant 2017, midseason 2018, and harvest 2018 soil samples. Total RN population density was greater in C1 and C2 than CS for midseason 2017 soil samples. Reproduction factor was greatest in CS and least in C2 in both 2017 and 2018. Plots receiving nematicide application in preplant 2018 had a significantly greater RN population density compared with untreated plots, but nematicide application did not affect the population density in any other season. There were significant irrigation by crop interactions for total RN population density in harvest 2017 and preplant 2018 soil samples. Irrigation effects varied significantly by cropping phase, but there was no consistent trend in these effects. Irrigation effects were significant in C1 and C2 for harvest 2017 soil samples, where total RN population density was greater in rainfed plots of C1 (6,151 RN/100 cm^3^ soil) and significantly lower in irrigated plots (3,513 RN/100 cm^3^ soil) while total RN population density was greater in irrigated plots of C2 (6,269 RN/100 cm^3^ soil) and significantly lower in rainfed plots (4,355 RN/100 cm^3^ soil). Irrigation effects were significant in CS and C2 for preplant 2018 soil samples, where total RN population density was greater in irrigated plots of CS (503 RN/100 cm^3^ soil) and significantly lower in rainfed plots (67 RN/100 cm^3^ soil) while total RN population density was greater in rainfed plots of C2 (11,559 RN/100 cm^3^ soil) and significantly lower in irrigated plots (7,845 RN/100 cm^3^ soil).

**Table 3.** Effects of irrigation, crop phase, and nematicide application on *Rotylenchulus reniformis* in 2017 and 2018.

Total *Rotylenchulus reniformis* population density																
	2017								2018							
	Pi^a^	Pm	Pf	Rf	Pi	Pm	Pf	Rf
ANOVA (*p*-values)																
Irrigation (I)	0.76	0.75	0.61	0.61	0.27	0.49	0.25	0.21
Crop phase (C)	< 0.01**	< 0.01**	< 0.01**	0.02*	< 0.01**	< 0.01**	< 0.01**	< 0.01**
I × C	0.35	0.46	0.02*	0.79	0.05*	0.82	0.39	0.16
Nematicide (N)	0.96	0.47	0.13	0.78	0.01**	0.22	0.13	0.78
I × N	0.89	0.70	0.22	0.62	0.10	0.72	0.53	0.16
C × N	0.18	0.75	0.84	0.50	0.29	0.90	0.74	0.41
I × C × N	0.67	0.80	0.65	0.90	0.18	0.84	0.53	0.34
Ratio of *Rotylenchulus reniformis* immature females and juveniles: males																
	2017								2018							
	Pi^a^	Pm	Pf		Pi	Pm	Pf	
ANOVA (*p*-values)																
Irrigation (I)	0.11	0.34	0.15		0.11	0.46	0.19	
Crop phase (C)	0.05*	0.18	0.01**		0.02*	0.28	0.03*	
I × C	0.93	0.38	0.92		0.10	0.36	0.43	
Nematicide (N)	0.95	0.61	< 0.01**		0.40	0.56	0.75	
I × N	0.18	0.84	0.08		0.37	0.43	0.95	
C × N	0.87	0.50	0.02*		0.72	0.64	0.83	
I × C × N	0.97	0.37	0.06		0.43	0.73	0.94	

**Notes:** ^a^Pi, Pm, and Pf are *p*-values for mean nematode population densities (per 100 cm^3^ soil) prior to planting, at midseason (52 and 56 days after planting in 2017 and 2018), and at harvest (150 and 151 days after planting in 2017 and 2018), respectively. Rf is reproduction factor (Pf/Pi). * and ** represent significant effects at *p* ≤ 0.05 and *p* ≤ 0.01, respectively.

Crop rotation significantly affected the ratio of RN immature females and juveniles to males in preplant and harvest soil samples in both 2017 and 2018 (Table 3). The ratio was greater in C2 than C1 and CS in preplant soil samples, but greater in CS than C1 and C2 in harvest soil samples. There was a significant crop by nematicide interaction on the ratio of RN immature females and juveniles to males in fall 2017. Nematicide effects were significant in CS, but not C1 or C2. In CS, the ratio of RN immature females and juveniles to males was greatest in nematicide-treated plots (14.0) and significantly lower in untreated plots (5.9).

### Ring nematode

**Figure 2:**
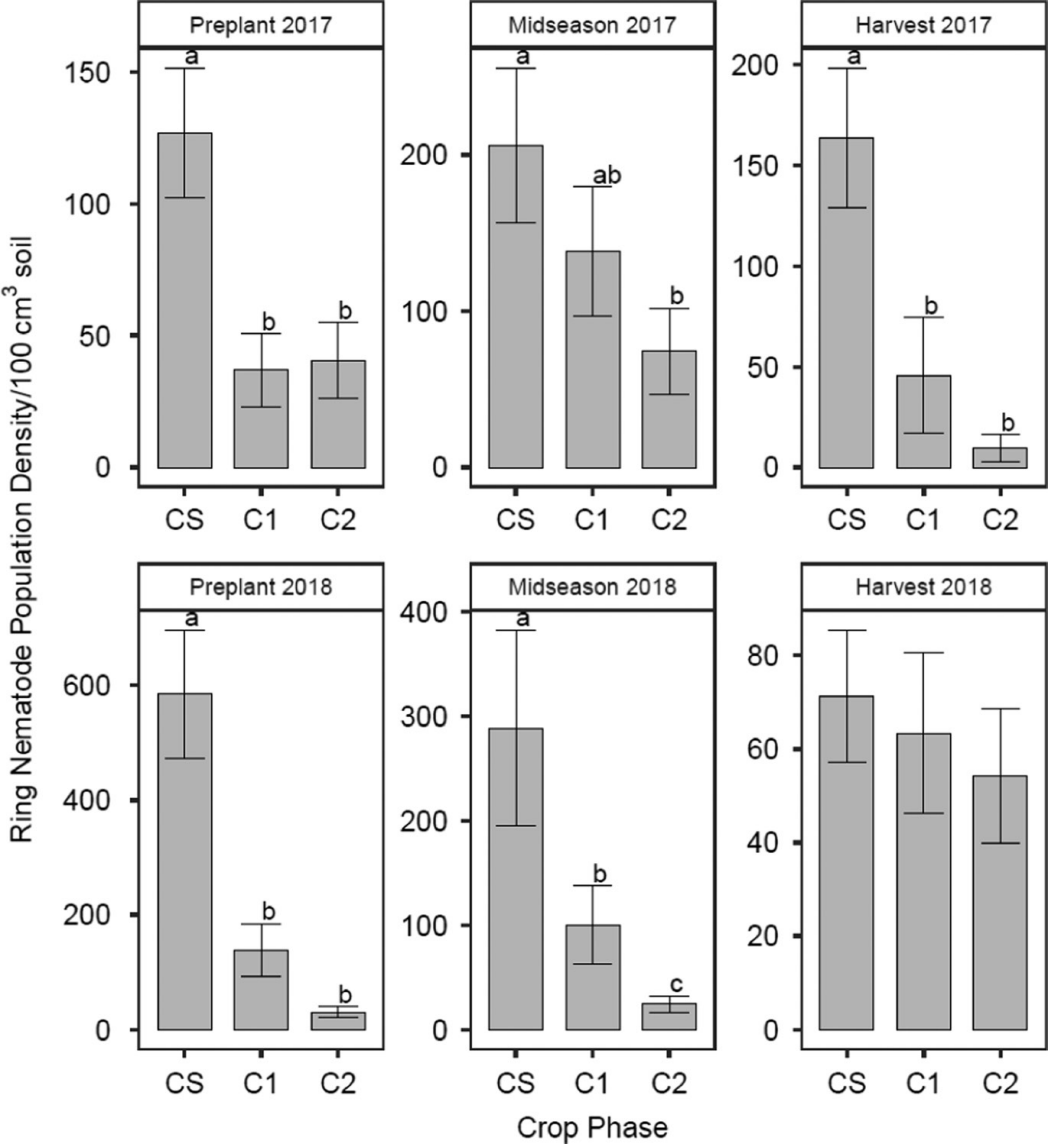
Ring nematode (*Mesocriconema ornatum*) population density (nematodes/100 cm^3^ soil) as influenced by crop phase in 2017 and 2018 soil samples. CS is the cotton phase of the sod-based rotation, C1 is first-year conventional cotton, and C2 is second-year conventional cotton. Different letters denote significant differences between crop phases (Fisher’s protected LSD, *p* ≤ 0.05).

Significant main effects of crop were observed in midseason 2017, harvest 2017, preplant 2018, and midseason 2018 soil samples (Fig. 2, Table 4). Ring nematode population density was greatest in CS and least in C1 and C2 in harvest 2017 and preplant 2018 soil samples. Ring nematode population density was greater in CS than C2 for midseason 2017 and midseason 2018 soil samples. Reproduction factor was greatest in C2 and least in CS in 2018. There was a significant irrigation by crop interaction for ring nematode population density in preplant 2017 soil samples. Irrigation effects were significant in CS, but not C1 or C2. In CS, ring nematode population density was greatest in irrigated plots (180 ring nematodes/100 cm^3^ soil) and significantly lower in rainfed plots (74 ring nematodes/100 cm^3^ soil). Ring nematode population density was greater in rainfed plots (292 ring nematodes/100 cm^3^ soil) than irrigated plots (210 ring nematodes/100 cm^3^ soil) in preplant 2018 soil samples. There was no statistical difference between nematicide-treated and untreated plots in any of the sampling dates.

**Table 4.** Effects of irrigation, crop phase, and nematicide application on *Mesocriconema ornatum* and *Helicotylenchus dihystera* in 2017 and 2018.

*Mesocriconema ornatum* population density														
	2017							2018						
	Pi^a^	Pm	Pf	Rf	Pi	Pm	Pf	Rf
ANOVA (*p* values)														
Irrigation (I)	0.14	0.19	0.29	0.96	< 0.01**	0.43	0.97	0.76
Crop phase (C)	< 0.01**	0.02*	< 0.01**	0.21	< 0.01**	0.05*	0.76	0.03*
I × C	0.04*	0.72	0.30	0.32	0.58	0.84	0.88	0.76
Nematicide (N)	0.71	0.66	0.53	0.81	0.31	0.22	0.30	0.13
I × N	0.68	0.42	0.15	0.98	0.78	0.10	0.27	0.74
C × N	0.97	0.54	0.52	0.55	0.15	0.42	0.97	0.13
I × C × N	0.34	0.87	0.63	0.42	0.99	0.25	0.55	0.76
*Helicotylenchus dihystera* population density														
	2017							2018						
	Pi^a^	Pm	Pf	Rf	Pi	Pm	Pf	Rf
ANOVA (*p* values)														
Irrigation (I)	0.33	0.60	0.93	0.31	0.06	0.28	0.22	0.36
Crop phase (C)	0.56	0.03*	0.19	0.90	0.07	0.15	0.04*	0.43
I × C	0.85	0.34	0.98	0.60	0.57	0.50	0.56	0.39
Nematicide (N)	0.02*	0.10	< 0.01**	0.75	0.02*	0.01**	< 0.01**	0.42
I × N	0.07	0.64	0.52	0.39	0.57	0.44	0.03*	0.50
C × N	0.42	0.01**	0.06	0.25	0.25	0.06	0.02*	0.28
I × C × N	0.64	0.09	0.29	0.37	0.40	0.15	0.09	0.24

**Notes:** ^a^Pi, Pm, and Pf are *p*-values for mean nematode population densities (per 100 cm^3^ soil) prior to planting, at midseason (52 and 56 days after planting in 2017 and 2018), and at harvest (150 and 151 days after planting in 2017 and 2018), respectively. Rf is reproduction factor (Pf/Pi). * and ** represent significant effects at *p* ≤ 0.05 and *p* ≤ 0.01, respectively.

### Spiral nematode

**Figure 3:**
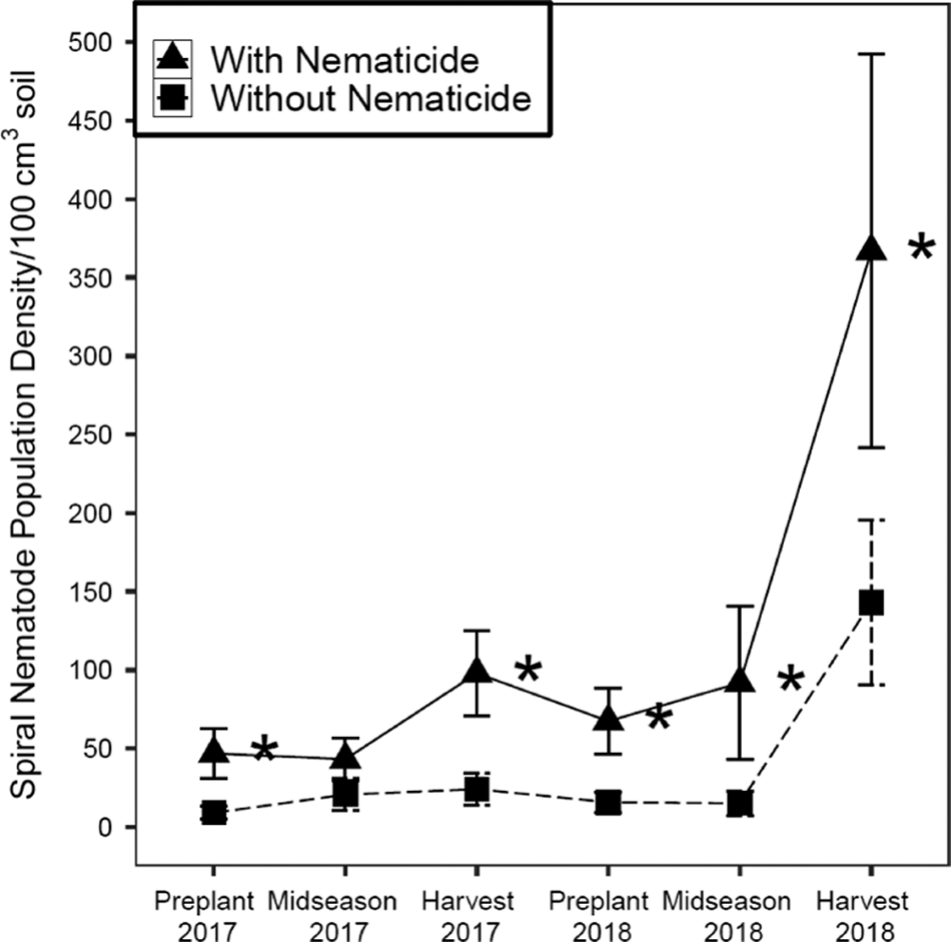
Spiral nematode (*Helicotylenchus dihystera*) population density (nematodes/100 cm^3^ soil) as influenced by nematicide application in 2017 and 2018 soil samples. With nematicide and without nematicide refer to the absence or presence of fluopyram, respectively. *indicates significant nematicide effect within the given season (*p* ≤ 0.05).

Significant main effects of crop were observed in midseason 2017 and harvest 2018 samples where spiral nematode population density was greatest in CS (Table 4). Significant main effects of nematicide were observed in preplant 2017, harvest 2017, preplant 2018, and midseason 2018 soil samples where spiral nematode population density was greater in nematicide-treated plots than untreated plots (Fig. 3). Nematicide effects were significant in CS, but not C1 or C2 for midseason 2017 and harvest 2018 soil samples. In CS midseason 2017 soil samples, spiral nematode population density was greatest in nematicide-treated plots (104 spiral nematodes/100 cm^3^ soil) and significantly lower in untreated plots (16 spiral nematodes/100 cm^3^ soil). In CS harvest 2018 soil samples, spiral nematode population density was greatest in nematicide-treated plots (775 spiral nematodes/100 cm^3^ soil) and significantly lower in untreated plots (211 spiral nematodes/100 cm^3^ soil). Irrigation effects on spiral nematode population density were significant in nematicide-treated plots, but not in untreated plots in harvest 2018 soil samples. In nematicide-treated plots, spiral nematode population density was greatest under irrigation (569 spiral nematodes/100 cm^3^ soil) and significantly lower in rainfed plots (165 spiral nematodes/100 cm^3^ soil).

### Cotton yield

**Figure 4:**
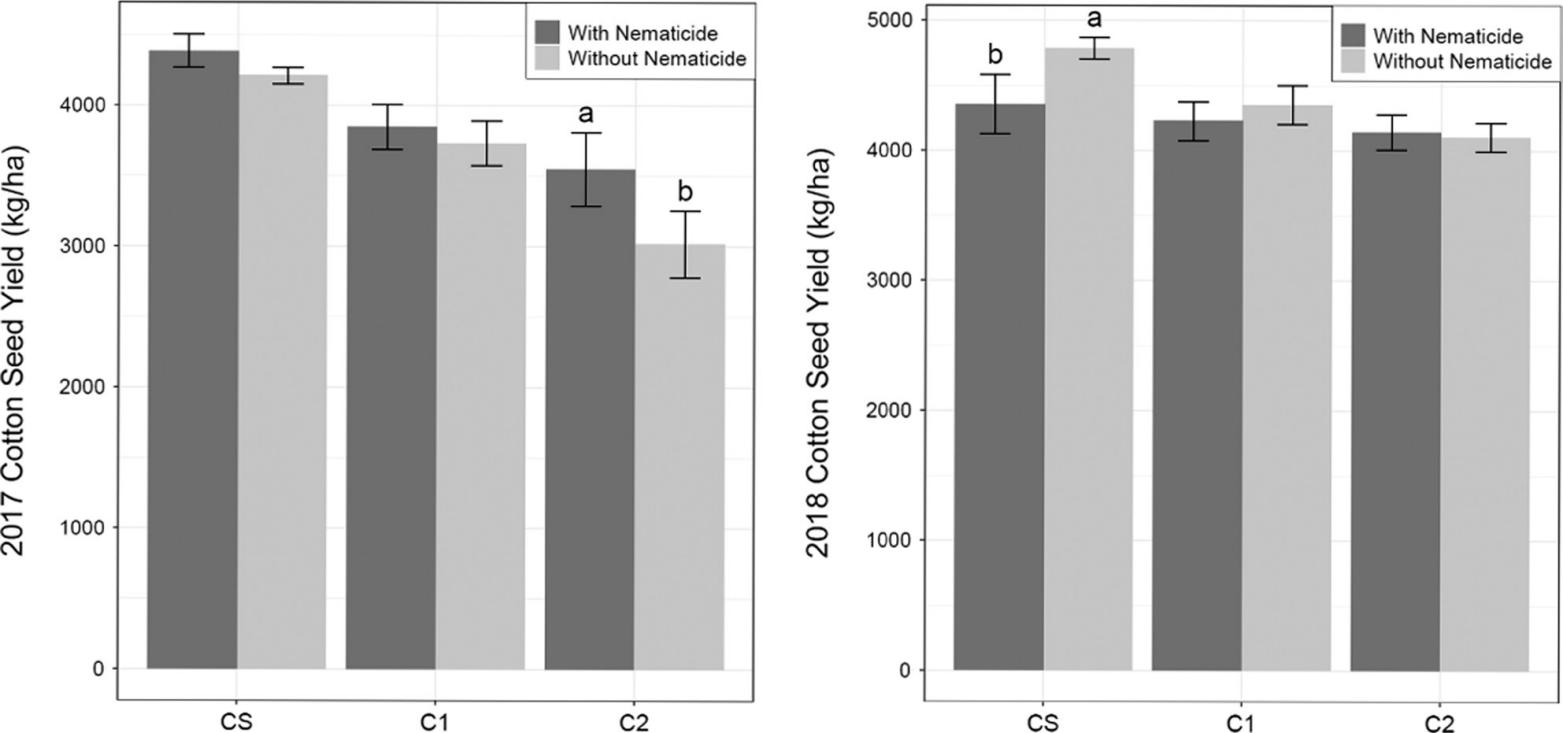
Cotton seed yield (kg/ha) in 2017 and 2018. CS is the cotton phase of the sod-based rotation, C1 is first-year conventional cotton, and C2 is second-year conventional cotton. With nematicide and without nematicide refer to absence or presence of fluopyram, respectively. Different letters denote significant differences between nematicide treatments within crop phase (Fisher’s protected LSD, *p* ≤ 0.05).

There was a significant crop by nematicide interaction for cotton seed yield in 2017 and 2018 (Table 5). In 2017, nematicide effects were significant in C2, but not CS or C1. In C2, cotton seed yield was greatest in nematicide-treated plots and significantly lower in untreated plots (Fig. 4). In 2018, nematicide effects were significant in CS, but not C1 or C2. In CS, cotton seed yield was greatest in untreated plots and significantly lower in nematicide-treated plots (Fig. 4). For cotton lint yield, significant main effects of crop and nematicide were observed in 2017 (Table 5). Cotton lint yield was greater in nematicide-treated plots than untreated plots. Cotton lint yield was greatest in CS and least in C2. There were no significant main effects of crop or nematicide observed on cotton lint yield in 2018.

**Table 5.** Effects of irrigation, crop phase, and nematicide application on cotton seed yield and lint yield (kg/ha).

	2017^a^		2018	
	Seed yield	Lint yield	Seed yield	Lint yield
ANOVA (*p* values)				
Irrigation (I)	0.50	0.32	0.06	0.31
Crop phase (C)	0.01**	0.01**	0.19	0.41
I × C	0.64	0.72	0.53	0.62
Nematicide (N)	< 0.01**	< 0.01**	0.03*	0.06
I × N	0.81	0.79	0.40	0.65
C × N	0.01**	0.09	0.05*	0.07
I × C × N	0.45	0.58	0.07	0.22

**Notes:** ^a^*p*-values for means at harvest (kg/ha). * and ** represent significant effects at *p* ≤ 0.05 and *p* ≤ 0.01, respectively.

## Discussion

Sod-based rotation managed RN populations better than conventional rotation. Our results were consistent with other research regarding greater RN population density in rotations with at least two years of cotton than those with more non-host crops (Cabanillas et al., 1999; Leach et al., 2012). A study by Davis et al. (2003) showed that effects of crop rotation on RN population density were undetectable by midseason when cotton was grown the following year. However, effects of crop rotation on RN population density at midseason were significant in our study, indicating that the populations were responding to specific crop phases, with the sod-based cotton phase resulting in fewer RN than conventional cotton phases. Possible explanations for differences between these studies are that our research was part of a long-term rotation (i.e. the non-host rotation was longer) and the aforementioned study utilized field sites with RN-resistant soybean in the crop rotation sequence. In this study, reproduction factor was greatest in sod-based cotton because initial RN population density was least in that phase. When growing a host crop, the lower the nematode population density is to begin with, the greater the reproductive potential, particularly following a non-host crop (Cabanillas et al., 1999). Therefore, bahiagrass and peanut helped reduce the initial population densities of RN in our study.

Little is known about RN sex ratios (i.e. proportions of females, juveniles, and males) throughout a growing season, although some studies estimate that these life stages exist in relatively equal numbers in the soil (Bird, 1983; Nakasono, 1966; Koenning et al., 2004). Nematode sex ratios may be influenced by environmental factors like food availability and temperature (Wright and Perry, 2006). In this study, RN sex ratios also followed this concept as the ratio of RN immature females and juveniles to males was greatest in second-year cotton before planting since that phase was the only one in which a host crop had been grown the previous year. At the end of the year, ratios of RN immature females and juveniles to males were greatest in sod-based cotton because the reproductive factor was greatest in that phase. Nakasono (2004) suggested that more RN males in relation to females could result from greater female death rate in the soil. In crop stress-inducing environments, such as rotations with non-host crops like SBR, the ratio of RN immature females and juveniles to males may be closer to one (i.e. equal numbers of males and immature females/juveniles) and in environments more favorable to the parasite, the ratio may be greater. It is important to understand RN sex ratios in order to estimate reproductive potential of RN populations in the soil. Another RN sex ratio study left out nematodes that are continuing their life cycles (i.e. maturing females) because they reside within cotton roots (Moore et al., 2010). We recognize information from sedentary females is missing in our RN sex ratio calculations, but the intent was to observe what happened with vermiform life stages in the soil. These ratios are important because they indicate RN reproductive favorability, and results of this study show that crop rotation systems affect these ratios reflecting RN reproductive capacity in the given environment.

Sod-based rotation increased populations of other plant-parasitic nematodes at the site, including spiral and ring nematodes. Because these nematodes have such broad host ranges, rotation, and cover crops may not be successful in managing their populations. Additionally, they are of minor importance in cotton production (Blasingame et al., 2002). It appears that sod-based rotation supported populations of both ring and spiral nematodes. Ring nematode population density was greatest in sod-based cotton, which was consistent with results from Tsigbey et al. (2009), where SBR reduced RN population density in peanut phases, but increased ring nematode population density compared to the conventional rotation. Our results differed in terms of spiral nematode population density, in which Tsigbey et al. (2009) reported a decrease in their numbers in sod-based rotation while we observed an increase. Bahiagrass is a poor host for RN but appears to be a suitable host for spiral nematodes (Andersen et al., 2016).

Fluopyram nematicide was not effective for managing plant-parasitic nematode populations at 50 or more days after planting in this study as it had no effect on ring nematode population density and increased spiral and RN population densities at various times compared with untreated control. Previous studies have found that fluopyram can be effective against other nematodes, including root-knot nematodes on vegetables (Hajihassani et al., 2019; Ji et al., 2019; Silva et al., 2019) and cotton (Roper, 2017) as well as a mixture of sting nematode (*Belonolaimus longicaudatus*) and spiral nematodes on turfgrass (Waldo et al., 2019). The concentration of fluopyram needed to paralyze nematodes in vitro is greater for RN than *M. incognita* (Faske and Hurd, 2015). Additionally, fluopyram has limited movement in xylem and is not systemic, so direct contact is required for nematode suppression (Faske and Hurd, 2015). Furthermore, non-fumigant nematicides tend to move poorly in soil, and RN is vertically distributed in the soil profile up to 1.5 m, so lack of fluopyram contact with RN deep in the soil profile may contribute to reduced fluopyram efficacy against RN in this trial (Lee et al., 2002; Westphal and Smart, 2003; Westphal et al., 2004; Robinson et al., 2005; Moore et al., 2010). Adjusting and maintaining the effective concentration of fluopyram in the soil may successfully manage plant-parasitic nematode populations in the field (Oka and Saroya, 2019). The results we observed in terms of spiral nematode population density increases with fluopyram application are consistent with findings in the turf industry relating to lance nematodes, which are in the same family (Hoplolaimidae) as RN. In this work, lance nematode population densities increased following fluopyram application in turfgrass (Crow, 2017). Therefore, additional research is needed to evaluate fluopyram rate efficacy for managing ring nematodes, spiral nematodes, and RN in conventional and sod-based rotations.

The main hypothesis in this study was ‘yield impact of nematicide application will be greater in conventional than sod-based rotation due to greater nematode pressure in conventional than sod-based rotation.’ This hypothesis was partially supported by our results. In 2017, nematicide protection from crop damage in terms of cotton seed yield was greater in conventional than sod-based rotation, which supports the hypothesis. However, in 2018 there was no benefit for cotton seed yield or lint yield with nematicide application in any rotation. Additionally, nematicide application increased lint yield equally in both rotations in 2017. This does not support our hypothesis. The positioning of the nematicide application in-furrow and its strong binding activity to soil suggests that fluopyram may only be effective in a relatively small zone (Faske and Hurd, 2015). Cotton in a conventional rotation may have smaller root systems with more roots located within the fluopyram-treated zone. Sod-based rotation results in cotton with a larger root system that likely developed deep roots (outside of the fluopyram-treated zone) and may explain the nematode populations observed in this study (Dourte et al., 2015). This larger root system may have allowed the plant to compensate for nematode damage. Furthermore, according to the Florida Automated Weather Network, cotton received adequate rainfall in both 2017 and 2018 (78 cm and 71.5 cm, respectively), so drought stress was not evident and helps explain the lack of observed yield effect in irrigated versus rainfed plots. Ultimately, nematicide application was ineffective at reducing nematode population densities and inconsistent in improving yield in this research.

Cotton yield was generally greater in SBR than conventional rotation and corresponded with decreased RN population density in SBR relative to conventional rotation. This suggests that RN damage is involved in the yield decrease observed in SBR, but other factors are also likely to be involved. The threshold set by Blasingame et al. (2002) was exceeded in our study, with fall RN population density greater than 1,000 nematodes/100 cm^3^ soil in both rotations. Preceding crops can affect the yield of following crops through soil fertility, allelochemicals, and other agronomic factors aside from nematodes and these factors likely contributed to the yield benefit of SBR (Jacobs et al., 2018). Our results clearly show that rotation was more consistent for managing RN and increasing yield than nematicide. Crop rotation will continue to be a paramount nematode management strategy.

Irrigation generally did not affect nematode population densities. Our results were inconsistent with other research looking at irrigation. Moore and Lawrence (2013) showed that RN population density was greater under irrigation, but their study evaluated soil types and not crop rotation or nematicide application. Additionally, population density of *Heterodera glycines* was greater in times of less rainfall (Bird et al., 2009). Our results indicate there was no significant yield benefit when cotton was irrigated. Yet, because we did not measure soil moisture in irrigated and non-irrigated plots, we cannot state with certainty if there were differences between the two regimes. One implication of irrigation in this study is that fluopyram must have adequate contact time in order to be effective, meaning timing of irrigation events need to be considered so as not to render the product ineffective (Faske and Hurd, 2015).

### Summary

In summary, there is evidence that nematicide application can be reduced in a sod-based rotation. Nematicide yield impacts varied by RN pressure. Population density of RN decreased in sod-based rotation and cotton yield was greater than the conventional cotton. Furthermore, sod-based rotation increased population densities of ring and spiral nematodes. Although nematicide application increased 2017 yield in second-year conventional cotton, it did not increase yield in sod-based cotton or first-year conventional cotton. This indicates that nematicide was more effective in the conventional rotation regarding yield. From a practical standpoint, a grower adopting a sod-based cotton rotation may be able to reduce or eliminate nematicide application while achieving desirable yield goals.
